# Internet-Delivered Cognitive Behavioral Therapy for Postsecondary Students: Randomized Factorial Trial for Examining Motivational Interviewing and Booster Lessons

**DOI:** 10.2196/40637

**Published:** 2022-09-07

**Authors:** Vanessa Peynenburg, Heather Hadjistavropoulos, David Thiessen, Nickolai Titov, Blake Dear

**Affiliations:** 1 Department of Psychology University of Regina Regina, SK Canada; 2 eCentre Clinic Department of Psychology Macquarie University Sydney Australia

**Keywords:** postsecondary students, transdiagnostic, boosters, motivational interviewing, internet-delivered cognitive behavioral therapy

## Abstract

**Background:**

Internet-delivered cognitive behavioral therapy (ICBT) can improve access to mental health care for students, although high attrition rates are concerning and little is known about long-term outcomes. Motivational interviewing (MI) exercises and booster lessons can improve engagement and outcomes in face-to-face cognitive behavioral therapy.

**Objective:**

This study aimed to examine the use of pretreatment MI exercises and booster lessons in ICBT for postsecondary students.

**Methods:**

In this factorial trial (factor 1: web-based MI before treatment; factor 2: self-guided booster lesson 1 month after treatment), 308 clients were randomized to 1 of 4 treatment conditions, with 277 (89.9%) clients starting treatment. All clients received a 5-week transdiagnostic ICBT course (the *UniWellbeing* course). Primary outcomes included changes in depression, anxiety, and perceived academic functioning from before treatment to after treatment and at the 1-month and 3-month follow-ups.

**Results:**

Overall, 54% (150/277) of students completed treatment and reported large improvements in symptoms of depression and anxiety and small improvements in academic functioning after treatment, which were maintained at the 1-month and 3-month follow-ups. Pretreatment MI did not contribute to better treatment completion or engagement, although small between-group effects favored MI for reductions in depression (Cohen *d*=0.23) and anxiety (Cohen *d*=0.25) after treatment. Only 30.9% (43/139) of students randomized to one of the booster conditions accessed the booster. Overall, no main effects were found for the booster. Subanalyses revealed that clients who accessed the booster had larger decreases in depressive symptoms (Cohen *d*=0.31) at the 3-month follow-up. No interactions were found between MI and the booster.

**Conclusions:**

Rather than offering MI before treatment, clients may experience more benefits from MI exercises later in ICBT when motivation wanes. The low uptake of the self-guided booster limited our conclusions regarding its effectiveness. Future research should examine offering a booster for a longer duration after treatment, with therapist support and a longer follow-up period.

**Trial Registration:**

ClinicalTrials.gov NCT04264585; https://clinicaltrials.gov/ct2/show/NCT04264585

## Introduction

### Background

An estimated one-third of college students worldwide meet the criteria for mental health disorders in any given year [1]. Depression, generalized anxiety disorder (GAD), and alcohol misuse are the most common mental health concerns among students and are associated with academic difficulties [2], distress [3], and increased risk of suicide [1]. During the COVID-19 pandemic, postsecondary students experienced many additional stressors that may contribute to poor mental health, including uncertainty about the future [4], adoption of new caregiving roles [5], limited opportunities for social contact [6], and strain on personal relationships [7]. Unfortunately, students face numerous attitudinal and structural barriers that prevent them from receiving treatment [8]. It is estimated that only approximately one-third of college students who meet the criteria for a mental health disorder in a given year receive some form of treatment [9].

Internet-delivered cognitive behavioral therapy (ICBT) is an effective alternative to face-to-face cognitive behavioral therapy (CBT) and may reduce the barriers that students face when trying to access treatment. In ICBT, clients receive structured web-based materials (eg, presentation slides, worksheets, and homework activities) based on cognitive behavioral strategies. Therapist-assisted ICBT courses typically involve weekly contact with a therapist either through secure messaging or telephone [10]. In a meta-analysis of internet interventions for university students, Harrer et al [11] found that interventions based on CBT had pooled effect sizes of *g*=0.29 for depression, *g*=0.39 for anxiety, and *g*=0.33 for stress. Overall, these effect sizes are smaller than what is reported in studies of ICBT within the general population [10], which suggests that strategies for improving the outcomes of ICBT among college and university students should be explored.

Another issue of ICBT for university students is high attrition rates [12]. In a study on predictors of outcomes in ICBT, it was found that younger clients were more likely to drop out of ICBT than older clients [13]. A possible explanation for high attrition rates among students is that they experience low motivation or ambivalence to change [14]. In face-to-face settings, low motivation and ambivalence to change can be addressed using motivational interviewing (MI) [15], and there is evidence for improved treatment acceptability, adherence [15], and treatment outcomes [16] in studies that combine MI with CBT [15]. Even single-session interventions involving MI have been found to produce larger effects than interventions that do not include MI in studies on reducing heavy drinking in college students [17]. Furthermore, brief MI interventions as short as 15 minutes can contribute to behavior changes [18]. Including a series of MI-based exercises at the beginning of ICBT may be beneficial to improving client outcomes.

The literature on adding MI to ICBT is limited, with only 2 studies exploring MI before clients initiate ICBT [19,20]. In 1 study, 108 clients with social anxiety were randomized to self-guided ICBT with or without a web-based MI lesson before treatment [20]. The MI lesson comprised reflective questions that encouraged clients to consider the short- and long-term costs and benefits of ICBT, the barriers they might encounter during ICBT, and the strengths they could draw on to overcome those barriers. Clients assigned to the MI lesson group were more likely to complete the full course of treatment than those who received ICBT alone (75% vs 56% completion), although no differences in outcomes were found between the groups. In another trial, 480 clients were randomized to either receive or not receive a pretreatment MI lesson before accessing ICBT [19]. The MI lesson comprised 3 videos and 5 web-based exercises with open-ended, reflective questions. Clients who received the MI lesson engaged in more change talk in their email exchanges with therapists and were enrolled in the course for more days than those who did not receive MI. However, there was no evidence that pretreatment MI contributed to better treatment completion rates or greater reductions in symptoms of depression and anxiety. The authors identified that motivation was high among clients before treatment and therefore speculated that benefits may not have been observed as clients did not generally experience low motivation.

To date, no studies have examined the inclusion of pretreatment MI in ICBT for postsecondary students, a group known to be at risk of lower engagement and poorer outcomes. Given the promising findings of including MI in face-to-face CBT [16] and preliminary findings for improved engagement in ICBT [19,20], it is worthwhile to explore the use of pretreatment MI to address high attrition rates among students.

Booster sessions represent another strategy with the potential to improve ICBT outcomes. In face-to-face CBT, booster sessions are used to remind clients of strategies learned during treatment and offer clients the opportunity to problem solve any barriers faced since completing treatment [21]. Booster sessions appear to help clients maintain treatment gains for a longer period of time than with treatment alone [21,22]. In the case of ICBT, there is some evidence showing that booster sessions after ICBT contribute to greater levels of overall functioning and delay the onset of relapse in clients with obsessive-compulsive disorder [23]. However, in another recent study of booster lessons with ICBT, the benefits of a booster session could not be adequately explored as uptake of the therapist-assisted booster lesson was low [24]. Overall, given the need to improve the engagement and outcomes of ICBT among postsecondary students, the benefits of booster lessons after ICBT are worthy of exploration.

### Objective

The purpose of this study was to examine the effects of including pretreatment MI and a self-guided booster offered 1 month after transdiagnostic ICBT for postsecondary students. In particular, we were interested in whether including pretreatment MI would affect treatment completion, treatment engagement, and outcomes compared with ICBT without pretreatment MI. We hypothesized that clients assigned to either of the conditions with pretreatment MI would be more likely to complete treatment and be more engaged during treatment (ie, greater number of log-ins and more messages sent to their therapist) than clients who did not receive the pretreatment MI exercises (ie, only received the standard ICBT course or the ICBT course with a self-guided booster lesson). The study of the benefits of pretreatment MI for symptom improvement was considered exploratory in this specific population, given the null findings from both previous ICBT studies regarding the benefits of pretreatment MI [19,20]. We were also interested in examining students’ use of a self-guided booster lesson and how the use of the booster would affect outcomes at the 3-month follow-up. It was hypothesized that clients assigned to the booster would have larger improvements than those not assigned to the booster.

## Methods

### Study Design

This study used a 2×2 factorial design (factor 1: pretreatment web-based MI; factor 2: self-guided booster lesson 1 month after treatment), and was registered as a clinical trial (ClinicalTrials.gov NCT04264585).

### Ethics Approval

The study was reviewed and received ethics approval from the University of Regina Research Ethics Board (REB: 2019-205).

### Setting

The study was conducted at a routine care ICBT clinic (the Online Therapy Unit), which offers ICBT free of charge to residents of Saskatchewan. Examining client outcomes in a routine care setting is important as these clients typically present with greater levels of comorbidity and more diversity than in early phase randomized controlled trials [25]. To identify small between-group effect sizes with a power of 80% and an α of .10, a minimum of 277 participants was required. An α of .10 has been suggested for optimization studies [26] and has been used in previous trials of ICBT to identify active factors [27]. Once a factor has been confirmed as important for improving outcomes in the confirmatory phase, a traditional α value of .05 can be used [26].

### Clients

Prospective clients could self-refer to the course using the Online Therapy Unit’s website. Clients found out about the *UniWellbeing* course through various sources, as described in the *Results* section. To be eligible, clients had to (1) be younger than 18 years, (2) self-report at least mild symptoms of depression or anxiety (ie, score ≥5) on the 9-item Patient Health Questionnaire (PHQ-9) [28] or 7-item Generalized Anxiety Disorder (GAD-7) questionnaire [29], (3) be enrolled at a postsecondary institution in the province of Saskatchewan, (4) have reliable access to a computer and the internet and feel comfortable using them, and (5) remain in Saskatchewan during the treatment period. Prospective clients were ineligible if they (1) had unmanaged psychosis, mania, or alcohol or drug problems; (2) were hospitalized in the previous year for mental health; (3) were at high suicide risk; or (4) did not meet the eligibility criteria listed previously.

### Intervention

All clients were offered the *UniWellbeing* course, which is a 5-week transdiagnostic, therapist-assisted ICBT course designed for postsecondary students. The course was developed at Macquarie University in Australia and has undergone several revisions to increase treatment completion [30,31]. In its current form, the *UniWellbeing* course comprises 4 lessons that are released over 5 weeks. Lessons are presented in a slideshow format and include case stories, downloadable lesson summaries, and homework exercises. The 4 lessons include information about the following: symptom identification and the CBT model, thought monitoring and thought challenging, symptoms of over- and underarousal and how they can be managed using breathing exercises and pleasant activity scheduling, avoidance or safety behaviors and graded exposure, and relapse prevention. Throughout the course, clients can access additional resources (ie, assertive communication, communication skills, emergency contact information, grief, managing beliefs, mental skills, and problem-solving), as well as 2 additional case stories that were created for this trial in response to therapist feedback (ie, COVID-19 and mature student case story). Clients receive automated messages to alert them about lessons as they become available and to remind them of how far along they should be in the course.

### Randomization and Factors

Clients who were accepted into the trial were randomized using REDCap (Research Electronic Data Capture; Vanderbilt University) to one of four treatment conditions (using block randomization) at the end of their telephone screen: standard *UniWellbeing*, MI+*UniWellbeing*, *UniWellbeing*+booster, or MI+*UniWellbeing*+booster.

#### MI Condition

Clients who were randomized to 1 of the 2 conditions that included MI completed a series of 5 web-based exercises (the *Planning for Change* lesson) before starting the main *UniWellbeing* course. The MI exercises were adapted from previous research [19,32] using previous client feedback, as well as input from 2 patient partners with lived experiences of anxiety or depression. Revisions included removing redundancy and improving the clarity of the exercises (eg, by including examples of strengths). Previous clients suggested that the MI exercises were too long, which could have negatively affected client motivation; therefore, the number of questions in each exercise was reduced.

In the trial by Soucy et al [19], clients completed the MI exercises 1 week before starting treatment, and the exercises were delivered on a separate platform from the main treatment, which prevented therapists from viewing client responses. In this trial, a decision was made to include the MI exercises directly on the treatment platform. All questions within each MI exercise were required for clients to access lesson 1 of the *UniWellbeing* course.

The exercises were based on common MI principles (ie, value clarification [33,34], importance ruler [33], looking back [33,34], confidence ruler [33], and looking forward [33]). Clients completed a series of open-ended questions as part of the exercises and received automated feedback to provide encouragement (eg, “You can do this. Your courage to reach out for help demonstrates that you have already started taking the steps to manage your anxiety and/or depression”)

#### Booster Lesson

Clients who were randomized to the booster condition were offered a self-guided booster lesson 1 month after completing the *UniWellbeing* cours*e*. The booster lesson comprised a summary of the key skills from the course, a section on maintaining motivation, and information about structured problem-solving and how it could be used to manage lapses. It was designed to reflect common content in face-to-face booster sessions [21]. The booster was presented in the same slideshow format as the core 4 lessons and included a printable summary with worksheets.

### Therapist Support

Each client was assigned to a therapist who provided weekly support during the 5-week course. Most of the therapist support was offered through personalized messages sent on the secure treatment portal on the same day each week. Therapists were instructed to spend 15 to 20 minutes per client each week. Telephone contact could be initiated if the client experienced a significant increase in symptoms of depression or anxiety (≥5 points on the PHQ-9 or GAD-7), if the client’s responses on the PHQ-9 or messages to their therapist suggested increased suicide risk, or if the client had not accessed the website for a week to encourage the client to continue working on the lessons. A total of 6 therapists provided support in the trial (n=2, 33% with backgrounds in psychology, and n=4, 67% in social work). Of the 6 therapists, 5 (83%) were registered with their respective regulatory colleges, and 1 (17%) was a supervised PhD student in clinical psychology. All 6 therapists received ICBT training, regular supervision, and auditing of their messages [35,36]. Therapists were encouraged to include the following in each message to the clients: express warmth and concern, provide feedback on weekly symptom questionnaires, highlight content from lessons, address client questions, reinforce skill acquisition and progress, manage risks, and remind clients of upcoming lesson content and their next check-in.

### Outcomes

#### Primary Outcome Measures

Primary outcome measures were administered before treatment, after treatment, and at the 1-month and 3-month follow-ups. Clients also completed these measures on a weekly basis during treatment as a way for therapists to monitor their symptoms.

##### 9-Item Patient Health Questionnaire

The PHQ-9 [28] comprises 9 self-report items to assess depressive symptoms over the past 2 weeks. Total scores range from 0 to 27, with a score of ≥10 often used as an indicator of clinically significant symptoms [28]. The PHQ-9 had good internal consistency (α=.83 to α=.86) in this study.

##### 7-Item Generalized Anxiety Disorder

The GAD-7 [29] comprises 7 self-report items to assess symptoms of GAD over the past 2 weeks. Total scores range from 0 to 21, and a score of ≥10 has been used to identify individuals with clinical levels of generalized anxiety [29]. The GAD-7 had good to excellent internal consistency in this study (α=.85 to α=.93).

##### Perceptions of Academic Functioning

The Perceptions of Academic Functioning (PAF) was created for this study and comprises 3 items related to academic functioning over the past week. Using a scale ranging from 0 to 10, clients were asked how well they felt they were able to attend their classes or lectures, complete academic tasks (eg, assignments, papers, laboratory classes, and readings), and absorb information from readings or lectures. The PAF had good internal consistency (α=.81 to α=.93), and items were summed to create a total score, with higher scores indicating better perceived functioning.

#### Secondary Outcome Measures

Secondary outcome measures were administered at various time points, as described in the following sections:

##### Sheehan Disability Scale

The Sheehan Disability Scale (SDS) [37] comprises 3 self-report items assessing mental health–related disability in the areas of work or school, social, and family life. Total scores range from 0 to 30, with higher scores indicating greater levels of impairment. The SDS was administered before treatment, after treatment, and at the 1-month and 3-month follow-ups and had acceptable to good internal consistency (α=.71 to α=.88).

##### Alcohol Use Disorder Identification Test

The Alcohol Use Disorder Identification Test (AUDIT) [38] is a 10-item self-report measure that includes questions on alcohol consumption. Total scores range between 0 and 40, with a score >20 warranting monitoring and brief intervention [39]. In this trial, the AUDIT was administered before and after treatment and had acceptable to good internal consistency (α=.77 to α=.81).

##### Drug Use Disorder Identification Test

The Drug Use Disorder Identification Test (DUDIT) [40] comprises 11 self-report items related to drug use. Total scores range between 0 and 44, and a score of ≥25 suggests the presence of drug dependence [40]. The DUDIT was administered before treatment and after treatment and had good internal consistency (α=.86 at both time points).

##### The 3-Item Change Questionnaire

The 3-Item Change Questionnaire (CQ-3) [41] comprises 3 items that focus on clients’ perceptions of their ability to make changes, the importance of making changes, and the level of commitment to making changes to their symptoms. All clients completed the CQ-3 before treatment, and clients assigned to MI also completed the CQ-3 after the MI exercises. The CQ-3 had poor internal consistency (α=.55 to α=.66).

##### Treatment Satisfaction Questionnaire

The Treatment Satisfaction Questionnaire [42] comprises 6 items to assess ICBT treatment satisfaction. Clients complete questions about whether they would refer the course to a friend, whether they thought the course was worth their time (*yes/no*), how satisfied they were with the overall treatment and quality of the course (1=*very dissatisfied* to 5=*very satisfied*), and the extent to which the course affected their confidence in managing their symptoms and motivation to seek help in the future (1=*greatly reduced* to 5=*greatly increased*).

### Analyses

**Figure 1:**
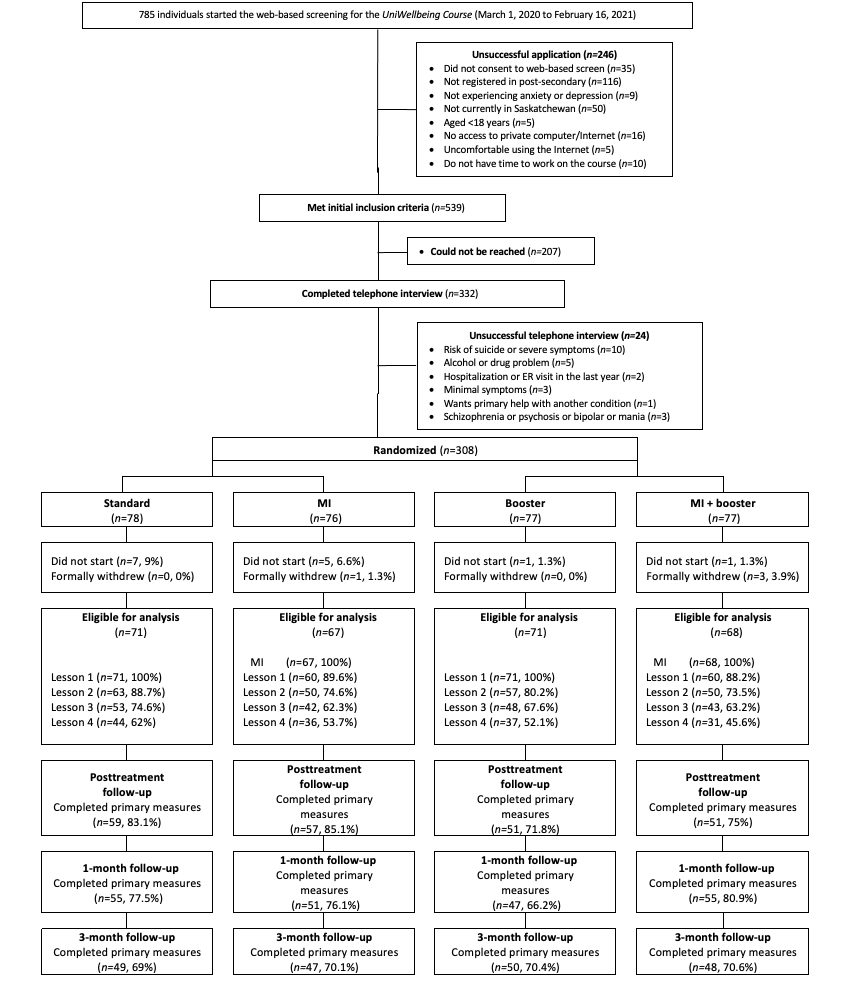
Patient flow from screening to the 3-month follow-up. ER: emergency room; MI: motivational interviewing.

Descriptive statistics were used to summarize client characteristics at intake, including demographics and scores on each of the primary and secondary outcome measures. Modified intention-to-treat (ITT) analyses [43] were used to generate replacement scores for missing data, which included only those clients who started treatment (Figure 1 shows the amount of missing data at each time point). A modified ITT was chosen to maintain consistency with the published trial of the *UniWellbeing* course [31], which allowed us to make more direct comparisons in effect sizes between studies, as opposed to a true ITT. In addition, true ITT analyses may be too conservative when examining effect sizes, as including clients who do not start treatment indicates little about the efficacy of the treatment [43]. A model was created that included 50 imputations and controlled for other values of the outcome measure, treatment condition, course completion, ethnicity, location, use of the booster, and interactions between pretreatment measures and the treatment condition [44-46]. Generalized estimation equations were used to examine changes in primary and secondary outcome measures across time points, consistent with past ICBT research [47,48]. Generalized estimation equation models used an exchangeable working correlation to account for within-subject variance. For the PHQ-9, GAD-7, PAF, and SDS, a gamma distribution model with a log link function was used, which can model improvements as proportional to pretreatment symptom severity and accommodate skewed response distributions [46]. For the AUDIT and DUDIT, the number of *0* value responses caused numerical issues; therefore, a more traditional Gaussian distribution with an identity link was used instead. At each of the 3 time points, pairwise comparisons were used to examine within-group and between-group effect sizes. Reliable improvement and reliable recovery were used as indicators of clinically significant change. Consistent with previous research, a reduction of 6 points on the PHQ-9 and 4 points on the GAD-7 was used as an indicator of reliable improvement [47,48]. Conversely, an increase of 6 points on the PHQ-9 and 4 points on the GAD-7 was an indicator of deterioration [47,48]. To meet the criteria for reliable recovery, clients had to score ≥10 on the PHQ-9 or GAD-7 before treatment, score <10 at the follow-up time point, and report at least a 6-point or 4-point reduction, respectively [47,48]. Finally, a series of ANOVAs and chi-square analyses were conducted to examine group differences in treatment completion, client engagement, and treatment satisfaction.

## Results

### Participant Flow and Characteristics

In this trial, 308 clients were randomized (Figure 1). To be considered eligible for analysis (277/308, 89.9%), clients had to consent to treatment and either start the MI exercises (MI conditions) or start lesson 1 (conditions that did not receive MI). The mean age of the sample was 23.73 (SD 5.95) years, and most of the sample were women (224/277, 80.9%) and White (201/277, 72.6%), had full-time student status (235/277, 85.5%), were living with someone (224/277, 80.9%), and were living within 1 of the 2 major cities in the province of Saskatchewan (191/277, 69%). Approximately half of the students (135/277, 48.7%) indicated that they were employed while completing their studies.

Before treatment, the mean scores on the PHQ-9 and GAD-7 were 14.49 (SD 5.77) and 13.71 (SD 4.76), respectively. Over two-thirds of clients scored above the clinical cutoff (≥10) on both the PHQ-9 and GAD-7, and only a small subset of clients did not score within the clinical range on either the PHQ-9 or the GAD-7 (34/277, 12.3%). The mean score on the PAF was 17.36 (SD 6.46). In terms of mental health history and service use, 37.9% (105/277) reported having a mental health diagnosis, 27.4% (76/277) reported having some form of mental health support at screening, and 36.5% (101/277) reported having taken psychotropic medication for anxiety or depression in the previous 3 months. The mean score for the CQ-3 was 24.05 (SD 4.22) before treatment, suggesting relatively high levels of motivation. Table 1 shows a full overview of the clients’ pretreatment characteristics.

**Table 1:** Pretreatment demographic and clinical characteristics by group (N=277).

Variable			All groups		Standard (n=71)		MI^a^ (n=67)		Booster (n=71)		MI+booster (n=68)
Age (years), mean (SD; range)			23.73 (5.95; 17-46)		23.57 (6.11; 18-44)		24.66 (6.58; 18-46)		23.35 (5.86; 17-43)		23.44 (5.28; 18-39)
**Gender, n (%)**											
	Man	45 (16.2)		10 (14.1)		6 (9.1)		12 (18.2)		17 (25)	
	Woman	224 (80.9)		57 (80.3)		58 (86.6)		58 (81.7)		51 (75)	
	Nonbinary	5 (1.8)		2 (2.8)		2 (3)		1 (1.5)		0 (0)	
	Prefer not to disclose or not listed	3 (1.1)		2 (2.8)		1 (1.5)		0 (0)		0 (0)	
**Marital status, n (%)**											
	Single or never married	113 (41.5)		31 (43.7)		22 (33.3)		31 (47)		29 (42.6)	
	Dating	107 (38.6)		26 (36.6)		24 (35.8)		29 (40.8)		28 (41.2)	
	Married or common law	31 (11.4)		8 (11.3)		13 (19.7)		5 (7.6)		5 (7.4)	
	Living with partner	18 (6.4)		4 (5.6)		3 (4.5)		5 (7.6)		6 (8.8)	
	Separated or divorced or widowed	8 (3)		2 (2.8)		5 (7.6)		1 (1.5)		0 (0)	
	Living with someone	224 (80.9)		55 (77.5)		56 (83.6)		57 (80.3)		56 (82.4)	
**Postsecondary institution, n (%)**											
	University of Regina	186 (67.1)		47 (66.2)		46 (70.1)		47 (66.2)		45 (66.2)	
	University of Saskatchewan	59 (21.3)		14 19.7 ()		11 (16.4)		18 (25.4)		16 (23.5)	
	Saskatchewan Polytechnic	10 (3.6)		1 (1.4)		6 (9.1)		1 (1.4)		2 (2.9)	
	Other	22 (8.1)		9 (12.7)		3 (4.5)		5 (7)		5 (7.7)	
**Student status, n (%)**											
	Full-time student	235 (85.5)		61 (87.1)		53 (79.1)		62 (87.3)		59 (88.1)	
	Part-time student	40 (14.5)		9 (12.9)		14 (20.9)		8 (12.7)		8 (11.9)	
**Year of studies, n (%)**											
	First-year undergraduate	67 (24.6)		19 (27.1)		14 (21.2)		21 (30.4)		13 (19.4)	
	Second-year undergraduate	57 (21)		13 (18.6)		17 (25.8)		8 (11.6)		19 (28.4)	
	Third-year undergraduate	62 (22.8)		18 (25.7)		17 (25.8)		16 (23.2)		11 (16.4)	
	Fourth-year undergraduate	44 (16.2)		10 (14.3)		7 (10.6)		15 (21.7)		12 (17.9)	
	Fifth or higher year undergraduate	18 (6.6)		3 (4.3)		7 (10.6)		4 (5.8)		4 (6)	
	Graduate or professional student	26 (8.8)		7 (10)		4 (6.1)		5 (7.2)		8 (12)	
International student, n (%)			10 (3.6)		3 (4.3)		2 (3)		1 (1.4)		4 (5.9)
English not the first language, n (%)			24 (8.7)		2 (2.8)		5 (7.5)		6 (8.6)		11 (16.2)
**Employment status, n (%)**											
	Paid work	135 (48.7)		39 (54.9)		32 (47.8)		30 (44.1)		30 (44.1)	
	Unemployed	142 (51.3)		32 (45.1)		35 (52.2)		38 (55.9)		38 (55.9)	
**Ethnicity, n (%)**											
	White	201 (72.6)		53 (74.6)		49 (73.1)		54 (76.1)		45 (66.2)	
	Indigenous	20 (7.2)		6 (8.5)		8 (11.9)		3 (4.2)		3 (4.4)	
	Asian	26 (9.4)		3 (4.2)		4 (6)		8 (11.2)		11 (16.2)	
	Other	30 (10.8)		9 (12.7)		6 (9)		6 (8.5)		9 (13.2)	
**Location^b^, n (%)**											
	Large city (>200,000)	191 (69)		49 (69)		44 (65.7)		52 (73.2)		46 (67.6)	
	Small to medium city	23 (8.3)		8 (11.3)		6 (9)		7 (9.9)		2 (2.9)	
	Small rural location (<10,000)	63 (22.7)		14 (19.7)		17 (25.4)		12 (16.9)		20 (29.4)	
**Referral source, n (%)**											
	Physician or medical professional	88 (31.9)		19 (27.1)		23 (34.3)		22 (31)		24 (35.3)	
	Web-based source (eg, website or email)	70 (25.4)		19 (27.1)		17 (25.4)		20 (28.2)		14 (20.6)	
	Counseling services	37 (13.4)		10 (14.3)		11 (16.4)		6 (8.5)		10 (14.7)	
	Friend or family member or employer	35 (12.7)		6 (8.6)		10 (14.9)		12 (16.9)		7 (10.3)	
	Presentation	12 (4.3)		5 (7.1)		2 (2.8)		2 (2.8)		3 (4.4)	
	Printed poster or media	4 (1.4)		4 (5.7)		0 (0)		0 (0)		1 (1.5)	
	Other	30 (10.9)		7 (10)		4 (6)		9 (12.7)		8 (11.8)	
**Mental health characteristics, n (%)**											
	Lifetime mental health service use	158 (57)		36 (50.7)		47 (70.1)		38 (53.5)		37 (54.4)	
	Lifetime hospitalization for mental health	22 (7.9)		3 (4.2)		9 (13.4)		7 (9.9)		3 (4.4)	
	Mental health diagnosis	105 (37.9)		27 (38)		32 (47.8)		27 (38)		19 (27.7)	
	Taking psychotropic medication in the past 3 months	101 (36.5)		20 (28.2)		29 (43.3)		23 (32.4)		29 (42.6)	
	Current mental health service use	76 (27.4)		19 (26.8)		23 (34.3)		12 (16.9)		22 (32.4)	
	Pretreatment GAD-7^c^ ≥10	216 (78)		58 (81.7)		49 (73.1)		55 (77.5)		54 (79.4)	
	Pretreatment PHQ-9^d^ ≥10	213 (76.9)		53 (74.6)		52 (77.6)		55 (77.5)		53 (77.9)	
	Pretreatment GAD-7 ≥10 and PHQ-9 ≥10	186 (67.1)		48 (67.6)		44 (65.7)		47 (66.2)		47 (69.1)	
	No clinical scores	34 (12.3)		8 (11.3)		10 (14.9)		8 (11.3)		8 (11.8)	
**Treatment expectations** **, mean (SD)**											
	CEQ^e^	21.16 (4.19)		20.63 (3.98)		21.91 (4.09)		20.90 (4.61)		21.34 (4.09)	
	CQ-3^f^	24.05 (4.22)		24.29 (3.64)		23.39 (4.51)		24.20 (4.46)		24.35 (4.41)	

^a^MI: motivational interviewing.

^b^Location is based on where the client was residing at intake.

^c^GAD-7: 7-item Generalized Anxiety Disorder.

^d^PHQ-9: 9-item Patient Health Questionnaire.

^e^CEQ: Credibility and Expectancy Questionnaire.

^f^CQ-3: 3-item Change Questionnaire.

### Primary Outcome Measures

The estimated marginal means, percentage reductions, and effect sizes for each of the primary outcome measures are presented in Table 2, separated by factor (MI vs no MI; booster vs no booster). The same information can be found in Multimedia Appendix 1 for each of the 4 treatment conditions (*UniWellbeing*, MI+*UniWellbeing*, *UniWellbeing*+booster, or MI+*UniWellbeing*+booster). Regardless of factors, there were statistically significant time effects for the PHQ-9, GAD-7, and PAF from before treatment to all subsequent measurement periods (after treatment and at the 1-month and 3-month follow-ups). Clients experienced large reductions on the PHQ-9 (Cohen *d*=1.28-1.48) and GAD-7 (Cohen *d*=1.46-1.72) from before treatment to after treatment, with improvements maintained at the 1-month (PHQ-9: Cohen *d*=1.27-1.37; GAD-7: Cohen *d*=1.29-1.51) and 3-month follow-ups (PHQ-9: Cohen *d*=1.22-1.31; GAD-7: Cohen *d*=1.19-1.31).

**Table 2:** Estimated marginal means, 95% CIs, percentage changes, and effect sizes (Cohen *d*d) for primary and secondary outcomes by factor (MI^a^ and booster) using pooled imputations.

Outcomes				Estimated marginal means, (SD)									Changes from pretreatment (%), 95% CI							Within-group effect sizes from pretreatment, 95% CI				
				Pretreatment		Posttreatment		1 month		3 months		To posttreatment			To 1 month		To 3 months		To posttreatment			To 1 month		To 3 months
**Primary outcomes**																								
	**PHQ-9^b^**																							
		MI	14.24 (5.56)		6.90 (4.24)		7.04 (4.92)		7.41 (4.78)		52 (46 to 57)			51 (44 to 57)		48 (42 to 54)		1.48 (1.21 to 1.75)			1.37 (1.10 to 1.63)		1.31 (1.05 to 1.58)	
		No MI	14.72 (5.97)		7.91 (4.54)		7.73 (5.01)		7.79 (5.31)		46 (41 to 52)			48 (41 to 54)		47 (41 to 53)		1.28 (1.02 to 1.54)			1.27 (1.01 to 1.52)		1.22 (0.97 to 1.48)	
		Booster	14.16 (5.16)		7.77 (4.41)		7.60 (5.00)		7.59 (4.78)		45 (40 to 51)			46 (40 to 53)		46 (40 to 53)		1.33 (1.07 to 1.59)			1.29 (1.03 to 1.55)		1.32 (1.06 to 1.58)	
		No booster	14.80 (6.33)		7.03 (4.41)		7.16 (4.95)		7.60 (5.33)		53 (47 to 58)			52 (45 to 58)		49 (42 to 55)		1.42 (1.16 to 1.69)			1.34 (1.08 to 1.60)		1.23 (0.97 to 1.48)	
	**GAD-7^c^**																							
		MI	13.51 (4.71)		6.10 (3.89)		6.78 (4.75)		7.17 (5.42)		55 (50 to 60)			50 (43 to 56)		47 (40 to 54)		1.71 (1.43 to 1.99)			1.42 (1.15 to 1.69)		1.25 (0.98 to 1.51)	
		No MI	13.88 (4.82)		7.14 (4.28)		7.11 (4.94)		7.51 (5.33)		49 (43 to 54)			49 (42 to 55)		46 (39 to 53)		1.47 (1.21 to 1.74)			1.38 (1.12 to 1.64)		1.25 (1.00 to 1.50)	
		Booster	13.66 (4.75)		7.12 (4.19)		7.34 (5.00)		7.59 (5.42)		48 (42 to 54)			46 (40 to 53)		44 (37 to 52)		1.46 (1.19 to 1.72)			1.29 (1.04 to 1.55)		1.19 (0.93 to 1.44)	
		No booster	13.73 (4.80)		6.12 (4.00)		6.57 (4.68)		7.10 (5.32)		55 (50 to 60)			52 (46 to 58)		48 (41 to 55)		1.72 (1.44 to 1.99)			1.51 (1.24 to 1.77)		1.31 (1.05 to 1.57)	
	**PAF^d^**																							
		MI	17.47 (6.27)		19.63 (7.30)		20.14 (6.73)		19.76 (7.62)		17 (7 to 28)			21 (11 to 32)		18 (7 to 30)		0.32 (0.08, 0.56)			0.41 (0.17 to 0.65)		0.33 (0.09 to 0.57)	
		No MI	17.24 (6.67)		19.24 (7.33)		20.38 (6.09)		19.12 (7.44)		16 (5 to 27)			25 (15 to 34)		15 (4 to 26)		0.28 (0.05 to 0.52)			0.49 (0.25 to 0.73)		0.27 (0.03 to 0.50)	
		Booster	17.29 (6.75)		19.81 (6.94)		20.52 (6.37)		19.41 (7.36)		20 (9 to 31)			25 (16 to 35)		17 (6 to 28)		0.37 (0.13 to 0.61)			0.49 (0.25 to 0.73)		0.30 (0.06 to 0.54)	
		No booster	17.43 (6.19)		19.05 (7.65)		19.99 (6.45)		19.48 (7.71)		13 (2 to 24)			20 (10 to 31)		16 (5 to 28)		0.23 (–0.01 to 0.47)			0.40 (0.17 to 0.64)		0.29 (0.06 to 0.53)	
**Secondary outcomes**																								
	**SDS^e^**																							
		MI	19.28 (6.70)		13.19 (6.79)		11.99 (7.27)		12.37 (7.46)		32 (25 to 38)			38 (31 to 45)		36 (29 to 43)		0.90 (0.65 to 1.15)			1.04 (0.79 to 1.30)		0.97 (0.72 to 1.22)	
		No MI	20.06 (5.75)		15.63 (7.00)		12.54 (7.09)		12.24 (7.38)		22 (16 to 28)			37 (31 to 44)		39 (32 to 46)		0.69 (0.45 to 0.93)			1.16 (0.91 to 1.41)		1.18 (0.93 to 1.43)	
		Booster	19.58 (6.22)		14.52 (6.53)		12.27 (7.25)		12.18 (7.13)		26 (19 to 32)			37 (30 to 44)		38 (31 to 45)		0.79 (0.55 to 1.03)			1.08 (0.83 to 1.33)		1.10 (0.85 to 1.35)	
		No booster	19.76 (6.26)		14.20 (7.45)		12.25 (7.12)		12.43 (7.70)		28 (21 to 35)			38 (31 to 45)		37 (30 to 44)		0.81 (0.56 to 1.05)			1.12 (0.86 to 1.37)		1.04 (0.79 to 1.29)	
	**AUDIT^f^**																							
		MI	4.59 (4.86)		3.08 (3.95)		—^g^		—		33 (18 to 48)			—		—		0.34 (0.10 to 0.58)			—		—	
		No MI	4.29 (4.06)		3.52 (3.90)		—		—		18 (2 to 34)			—		—		0.19 (–0.04 to 0.43)			—		—	
		Booster	4.30 (4.28)		3.28 (3.86)		—		—		24 (7 to 40)			—		—		0.25 (0.01 to 0.48)			—		—	
		No booster	4.58 (4.65)		3.32 (4.00)		—		—		28 (12 to 43)			—		—		0.29 (0.05 to 0.53)			—		—	
	**DUDIT^h^**																							
		MI	2.32 (5.10)		1.95 (4.72)		—		—		16 (20 to 52)			—		—		0.07 (–0.16 to 0.31)			—		—	
		No MI	2.90 (6.04)		2.36 (5.21)		—		—		19 (–12 to 49)			—		—		0.10 (–0.14 to 0.33)			—		—	
		Booster	2.52 (5.09)		1.66 (3.65)		—		—		34 (8 to 60)			—		—		0.19 (–0.04 to 0.43)			—		—	
		No booster	2.70 (6.09)		2.66 (6.00)		—		—		2 (–36 to 40)			—		—		0.01 (–0.23 to 0.24)			—		—	

^a^MI: motivational interviewing.

^b^PHQ-9: 9-item Patient Health Questionnaire.

^c^GAD-7: 7-item Generalized Anxiety Disorder.

^d^PAF: Perceptions of Academic Functioning.

^e^SDS: Sheehan Disability Scale.

^f^AUDIT: Alcohol Use Disorder Identification Test.

^g^The AUDIT and DUDIT were only administered before treatment and after treatment; thus, data are not available for the percentage change and effect sizes at the 1-month and 3-month follow-ups.

^h^DUDIT: Drug Use Disorder Identification Test.

A main effect was found for MI on the PHQ-9 (between-group Cohen *d*=0.23, 95% CI −0.01 to 0.47; *P*=.06) and GAD-7 (between-group Cohen *d*=0.25, 95% CI 0.02-0.49; *P*=.04) after treatment, whereby clients who were randomized to the MI condition had larger reductions in both measures from pretreatment to posttreatment time points. Between-group differences were no longer significant at the 1-month or 3-month follow-ups for the PHQ-9 (*P*=.25-.52) or GAD-7 (*P*=.57-.60). No significant between-group differences were found for the PAF at any of the 3 time points (*P*=.48-.75).

No main effects were found for those assigned to the booster versus those who were not assigned to any of the primary (*P*=.45-.99) or secondary measures (*P*=.03) at the 3-month follow-up. Owing to low booster use, subanalyses compared clients who did and did not access the booster and found the main effects in favor of accessing the booster on the PHQ-9 (*P*=.09) and PAF (*P*=.02). Clients who accessed the booster had larger improvements in depression (between-group Cohen *d*=0.31, 95% CI −0.05 to 0.67) and perceived academic functioning (between-group Cohen *d*=0.42, 95% CI 0.06-0.78) at the 3-month follow-up. Of note, clients who accessed the booster had higher perceived academic functioning before treatment (between-group Cohen *d*=0.33, 95% CI −0.03 to 0.69; *P*=.08), after treatment (between-group Cohen *d*=0.47, 95% CI 0.11-0.83; *P*=.01), and the 1-month follow-up (between-group Cohen *d*=0.33, 95% CI −0.03 to 0.69; *P*=.07); thus, it is likely that high perceived academic functioning is a predictor of booster use. In contrast, between-group effects on the PHQ-9 did not emerge until after the booster was offered. Between-group effects were not significant for the GAD-7 (*P*=.21) or SDS (*P*=.61) at the 3-month follow-up. Table 3 includes additional details on the between-group effects.

**Table 3:** Between groups effect sizes (Cohen *d*) for primary and secondary outcomes based on MI^a^ and booster factors using pooled imputations.

Outcomes				After treatment, Cohen *d* (95% CI)		1-month follow-up, Cohen *d* (95% CI)		3-month follow-up, Cohen *d* (95% CI)
**MI**								
	**Primary outcomes**							
		PHQ-9^b^	0.23 (−0.01 to 0.47)		0.14 (−0.10 to 0.37)		0.08 (−0.16 to 0.31)	
		GAD-7^c^	0.25 (0.02 to 0.49)		0.07 (−0.17 to 0.30)		0.06 (−0.17 to 0.30)	
		PAF^d^	0.05 (−0.18 to 0.29)		−0.04 (−0.27 to 0.20)		0.09 (−0.15 to 0.32)	
	**Secondary outcomes**							
		SDS^e^	0.35 (0.12 to 0.59)		0.08 (−0.16 to 0.31)		−0.02 (−0.25 to 0.22)	
		AUDIT^f^	0.11 (−0.12 to 0.35)		—^g^		—	
		DUDIT^h^	0.08 (−0.15 to 0.32)		—		—	
**Booster**								
	**Primary outcomes**							
		PHQ-9	−0.17 (−0.40 to 0.07)		−0.09 (−0.32 to 0.15)		0.00 (−0.23 to 0.24)	
		GAD-7	−0.24 (−0.48 to −0.01)		−0.16 (−0.39 to 0.08)		−0.09 (−0.33 to 0.14)	
		PAF	0.10 (−0.13 to 0.34)		0.08 (−0.15 to 0.32)		−0.01 (−0.25 to 0.23)	
	**Secondary outcomes**							
		SDS	−0.05 (−0.28 to 0.19)		0.00 (−0.24 to 0.23)		0.03 (−0.20 to 0.27)	
		AUDIT	0.00 (−0.23 to 0.24)		—		—	
		DUDIT	0.20 (−0.03 to 0.44)		—		—	

^a^MI: motivational interviewing.

^b^PHQ-9: 9-item Patient Health Questionnaire.

^c^GAD-7: 7-item Generalized Anxiety Disorder.

^d^PAF: Perceptions of Academic Functioning.

^e^SDS: Sheehan Disability Scale.

^f^AUDIT: Alcohol Use Disorder Identification Test.

^g^The AUDIT and DUDIT were only administered before treatment and after treatment; thus, data are not available for the percentage change and effect sizes at the 1-month and 3-month follow-ups.

^h^DUDIT: Drug Use Disorder Identification Test.

### Secondary Outcome Measures

Table 2 also includes details on the estimated marginal means, percentage reductions, and effect sizes for each of the secondary measures separated by factor. Multimedia Appendix 1 shows an overview of this information, separated into the 4 treatment conditions. Significant time effects were found for the SDS, regardless of factor. Table 3 summarizes the between-group effect sizes.

For MI, a small between-group effect was found after treatment, such that clients who received MI had larger improvements on the SDS than clients who did not receive MI (between-group Cohen *d*=0.35, 95% CI 0.12-0.59). At the 1-month (Cohen *d*=−0.24 to 0.23) and 3-month follow-ups (Cohen *d*=−0.20 to 0.27), these differences were no longer present, and there were large within-group effect sizes for improvements on the SDS, regardless of factor (Cohen *d*=1.02-1.25) and the 3-month follow-up (Cohen *d*=0.97-1.18). No main effect was found for MI for the AUDIT (*P*=.35) or DUDIT (*P*=.49) after treatment, and these measures were not administered during follow-up.

No main effects for randomization to the booster were found for the SDS (*P*=.78). Similarly, the subanalysis comparing those who accessed the booster and those who did not access the booster failed to find group differences (*P*=.61). The AUDIT and DUDIT were not administered at the 3-month follow-up.

### Clinical Significance

After treatment, 47.7% (132/277) of all clients met the criteria for reliable recovery, 60.3% (167/277) met the criteria for reliable improvement, 1.9% (5/277) met the criteria for deterioration, and 37.9% (105/277) met the criteria for no change on the PHQ-9. For the GAD-7, the rate of reliable recovery was 56.6% (157/277), the rate of reliable improvement was 75.5% (209/277), the rate of deterioration was 2.2% (6/277), and the rate of no change was 22% (61/277). At all time points, no significant main effects were found for MI or booster (*P*=.13-.99).

### Treatment Engagement

Of the clients in one of the MI conditions, 88.9% (120/135) completed the MI exercises and started lesson 1 (MI: 60/67, 90%; MI+booster: 60/68, 88%). Overall, 66.8% (183/277) of the clients accessed at least three of the four lessons, and 54.2% (150/277) accessed all 4 lessons within the 5 weeks of treatment. Of the clients who were randomized to booster, 30.9% (43/139) accessed the booster. Across conditions, clients logged in an average of 12.95 (SD 9.15) times, received an average of 5.23 (SD 0.83) emails from their therapists, and sent an average of 1.98 (SD 1.71) emails to their therapists. There was an average of 29.05 (SD 19.16) days between the clients’ enrollment date and their last log-in to the treatment portal. No main effects were found for MI on any measure of treatment engagement (*P*=.11-.75). Similarly, the main effects for the booster were not significant (*P*=.21-.95). Subanalyses comparing those who accessed the booster and those who did not found that clients who accessed the booster had a greater number of days between enrollment and their last visit to the treatment portal (mean 53.19, SD 15.70 vs mean 34.49, SD 20.65; *F*_1,267_=3.41; *P*=.07) and received a greater number of phone calls from their therapist (mean 0.51, SD 0.87 vs mean 0.44, SD 0.51; *F*_1,273_=3.21; *P*=.07). Therapists spent an average of 110.55 (SD 43.66) minutes monitoring client progress and supporting each client, with no significant differences found for mean time per client (*F*_3,274_=11.14; *P*=.33).

### Treatment Satisfaction

Clients reported high rates of satisfaction overall, with 82.3% (158/192), 85.5% (165/193), and 84.5% (163/193) reporting that they were satisfied or very satisfied with the treatment, the treatment platform, and the lessons and do-it-yourself guides, respectively. Most clients felt that the treatment was worth their time (171/192, 89.1%) and that they would recommend it to a friend (176/193, 91.2%). Furthermore, 82.9% (160/193) of clients reported that their motivation to seek help if needed in the future either increased or greatly increased, and 76.2% (147/193) felt that their confidence in their ability to manage their symptoms either increased or greatly increased. No significant differences were found between treatment conditions on any of the treatment satisfaction measures (*P=*.37-.83).

## Discussion

Although ICBT is an effective treatment option for postsecondary students experiencing symptoms of anxiety or depression [11], there are concerns about treatment completion and outcomes in this population. This study extends previous work on ICBT for postsecondary students by examining the inclusion of pretreatment MI exercises and a self-guided booster lesson offered 1 month after treatment.

### Impact of MI Exercises

Some benefit was found for the inclusion of pretreatment MI on symptoms of depression, anxiety, and overall functioning after treatment. Clients who were randomized to one of the MI conditions reported larger improvements in symptoms of depression, anxiety, and overall functioning from before treatment to after treatment than those of clients who were not assigned MI. No benefit for MI was found at either the 1-month or 3-month follow-up; thus, it appears that pretreatment MI may only result in temporary benefits compared with ICBT without MI. MI did not contribute to higher rates of treatment completion or greater engagement (ie, more log-ins to the website, more days enrolled in the course, or more client messages sent to therapists). Findings from this trial replicate those of a previous trial that examined pretreatment MI before an 8-week ICBT program [19] with 1 exception—we found some evidence for pretreatment MI contributing to greater symptom improvement for depression, anxiety, and overall functioning after treatment, whereas the previous trial found no benefits.

An explanation for why the MI exercises improved some outcomes despite no observable increase in treatment engagement is that the MI exercises helped elicit more change talk from the clients. Change talk was not examined directly in this study; however, a previous trial found that clients who completed pretreatment MI exercises included more change talk statements in messages with therapists than those who did not complete the exercises, despite no differences in treatment completion rates between the groups [19]. Other studies have described how the inclusion of MI in ICBT can lower client resistance to treatment [49]. Clients who completed the MI exercises may have been more engaged with the lessons and homework activities they completed, although they did not complete more lessons overall.

Pretreatment MI may not have led to higher treatment completion rates in both this trial and that of Soucy et al [19] as clients already have relatively high mean pretreatment motivation (CQ-3 scores in the study by Soucy et al [19] 25.44-25.59; CQ-3 scores in this trial 23.39-24.35). It had been hypothesized that students experience low levels of motivation in ICBT; however, the findings of this trial suggest otherwise, and it may be that the MI exercises are not relevant for many clients. Within the literature on face-to-face CBT, it has been reported that integrating MI throughout CBT can lead to higher rates of recovery in GAD at the 1-year follow-up than with CBT alone [50]. Thus, it may be worthwhile for future research to explore the integration of MI throughout the course of ICBT and the targeting of MI among less-motivated clients.

### Impact of Booster

The inclusion of a self-guided booster lesson in ICBT for postsecondary students has not been previously examined; thus, no hypotheses were made regarding the proportion of clients who would make use of a booster. Overall, there were no significant differences between those assigned to the booster and those who were not. The lack of differences is likely, in part, related to the low use of the booster lesson. Booster use in this study was lower (43/134, 31.9%) than that in previous trials of boosters in ICBT (32/47, 68% in the study by Andersson et al [23] and 114/223, 51.5% in the study by Hadjistavropoulos et al [24]), although both these trials included therapist support during the booster, which may have been more favorable to clients than a self-guided booster lesson. There was evidence for lower symptoms of depression at the 3-month follow-up among clients who accessed the booster than among those who did not access the booster, which was in contrast with the findings of a recent trial of a therapist-assisted booster following ICBT [24]. It is possible that clients who felt that they were managing their academic studies well were more likely to believe that they had the time to review the booster lesson. In addition, clients who benefited from the first 5 weeks of treatment might have been more likely to access the booster, as it has been found that initial success with an intervention predicts booster outcomes [51]. Future research should explore the impact of boosters under varying conditions (eg, different periods, contents, and levels of support).

### Overall Outcomes

Although findings related to MI and the booster condition were limited and completion rates were slightly above 50%, across the treatment conditions, clients experienced large reductions in both depression (Cohen *d*=1.25-1.67) and anxiety (Cohen *d*=1.42-2.01) after treatment, replicating past findings on the *UniWellbeing* course in another context. The slightly larger effect sizes in this trial may be attributed to the fact that the clients in this trial had higher symptom severity before treatment. It is possible that clients in our trial experienced an exacerbation of pre-existing symptoms of depression and anxiety as a result of the COVID-19 pandemic [52], which may have contributed to their slightly higher mean scores on the PHQ-9 and GAD-7 before treatment. Clients who start ICBT with more severe symptoms have greater symptom improvement than those with less severe symptoms [13]. Furthermore, Dear et al [31] reported on subanalyses of clients who started with moderate to severe scores on the PHQ-9 and GAD-7 and found larger effect sizes for depression (Cohen *d*=1.42-1.97) and anxiety (Cohen *d*=1.93-2.13) among these clients.

Treatment completion rates were similar between this study (150/277, 54.1%) and a previous trial (59%) [31] and were even more similar when comparing this study with the clients who self-referred (53%) to the *UniWellbeing* course in the previous trial of *UniWellbeing* [31]. In this trial, we found that clients logged in more days on average than in the previous trial (12.95 vs 8.70 log-ins) [31]. The previous trial did not report on the average number of days that clients were enrolled in the course or the average number of messages that clients sent to therapists; therefore, a direct comparison cannot be made between the 2 trials.

Previous trials of the *UniWellbeing* course [30,31] did not include subjective measures of academic functioning; therefore, the inclusion of the PAF in this trial is a unique contribution. Although it was hypothesized that there would be moderate effects for improvements on the PAF, we found small effects from pretreatment to posttreatment across the 4 treatment conditions. The studies included in the meta-analysis conducted by Harrer et al [11] either used measures of overall functioning or relied on a single objective indicator of academic functioning, such as grade-point average. Therefore, a direct comparison between the findings on subjective academic functioning in this trial and the findings of Harrer et al [11] is not possible. The finding that clients only experienced small improvements in perceived academic functioning should be considered within the context of the COVID-19 pandemic. Students faced considerable uncertainty about classes, lectures, examination formats, and the future of their academic careers [53], and this may factor into their overall ability to cope with academic pressures while completing ICBT.

It is challenging to compare the findings of this trial with the overall effects from meta-analyses of ICBT for postsecondary students [11] because of the heterogeneity of the studies included. Studies varied substantially in terms of guidance (eg, self-guided vs therapist-assisted), recruitment (eg, psychology participant pool vs clinical sample), and treatment modality (eg, website vs app). Overall, the effect sizes for improvements in depression and anxiety appeared larger in this trial than in the meta-analysis [11] and may be explained by the inclusion of weekly therapist support in this trial [54] and the requirement that clients have at least mild symptoms at intake [13].

Although the *UniWellbeing* course has been compared with a wait-list control in the past [30], a control group was not included in this study, and it is likely that some of the improvements in symptoms were because of a regression to the mean. It has been reported that 20% of students with major depressive disorder experience remission at the end of a 9-week observation period in the absence of treatment [55]; therefore, it would be expected that a proportion of students in this trial may have improved without ICBT.

### Limitations and Future Directions

There were several limitations to this study, which can inform future trials of ICBT for postsecondary students. One of the limitations was that the MI component was only offered before treatment, which may not have been the most beneficial time to offer MI to clients as clients may be starting treatment with high levels of motivation. In future trials of the *UniWellbeing* course, clients could be offered an MI resource that they could access at any point during the course, as opposed to before treatment. If therapists note client disengagement, ambivalence, or resistance, they could direct the client to this resource, similar to how therapists direct clients to additional resources (eg, sleep and assertive communication) as part of their practice when delivering ICBT. Soucy et al [19] found that the MI exercises resulted in an increase in client motivation and *change talk*; thus, client use of an MI resource during ICBT could facilitate greater treatment completion when offered at a more appropriate time.

Future studies could also examine a *blended* version of the *UniWellbeing* course, whereby the therapist contact remains unchanged for the 4 core lessons, but the client has the opportunity to schedule an appointment with their therapist via telephone or secure video call to review the MI exercises. A telephone or video call would enable the client to receive direct feedback from their therapist and would provide opportunities for the therapist to respond in the “spirit” of MI (ie, emphasizing collaboration with the client, evoking change, and emphasizing the autonomy of the client to initiate change) [33]. A longer follow-up period (eg, 6 or 12 months after treatment) would allow for a better understanding of the long-term effects of MI on symptom reduction. It is also possible that treatment noncompletion was not related to motivation but instead to students having preferences for different treatment doses. Future studies could offer students the choice between a brief, standard (5-week), or extended version of the ICBT course to align with student preferences.

A limitation of the booster lesson was that it was offered only 1 month after treatment completion. Some clients may not have felt that they needed a booster lesson soon after treatment, which likely contributed to the low overall uptake of the booster. Among those who used the booster, there was preliminary evidence suggesting that the booster was associated with larger reductions in depression at the 3-month follow-up; however, these subanalyses were underpowered because of low uptake. Furthermore, we are unable to comment on the longer-term impacts of the booster lesson on symptoms of depression and anxiety, as well as subjective academic functioning, given that the final outcome measures were administered at the 3-month follow-up. Andersson et al [23] found that boosters in ICBT can help reduce relapse rates of obsessive-compulsive disorder up to at least the 1-year follow-up; therefore, it would be worthwhile to examine the impact of a self-guided booster lesson over a longer follow-up period than used in this trial.

It should be noted that all the clients enrolled in this trial started treatment during the COVID-19 pandemic. It is possible that clients experienced regression to the mean in terms of their symptom severity as they became accustomed to COVID-19 public health restrictions and the impact on their academic studies. As this is the first trial of the *UniWellbeing* course in Saskatchewan, we do not have a comparison sample to use as a benchmark. Only 16.2% (45/277) of our sample identified as men; thus, future studies should attempt to recruit a more balanced representation of genders. A further limitation is that a modified ITT approach was used, which eliminated any data from the 10.1% of the clients who did not start treatment. Finally, the PAF was designed for this study and requires further validation beyond internal consistency in future research.

### Strengths

A notable strength of this study is that we were able to replicate the findings of a previous trial on the *UniWellbeing* course [31] in terms of large reductions in symptoms of depression and anxiety that were maintained at the 3-month follow-up. This replication is important as it provides evidence for the generalizability of the initial findings in a different country (ie, Canada) and within the context of the COVID-19 pandemic. An additional strength is the use of a factorial design, which allowed for the concurrent examination of both pretreatment MI and a self-guided booster lesson within a single trial. To the best of our knowledge, this combination of factors has not been examined in previous trials of ICBT for postsecondary students. This trial also provides useful information about students’ interest in, and uptake of, a self-guided booster lesson following ICBT.

### Conclusions

The findings from this factorial trial provide evidence for the efficacy of a 5-week transdiagnostic ICBT course for postsecondary students. Large effect sizes were found for reductions in symptoms of depression and anxiety, and small effect sizes were found for improvements in perceived academic functioning, with changes maintained up to the 3-month follow-up. There was some evidence for the benefit of pretreatment MI in improving depression, anxiety, and disability outcomes after treatment; however, no benefit was found for treatment completion or engagement. No main effects were found for the inclusion of a booster. However, although the booster was used by less than one-third of clients, there was some evidence for improved depression outcomes at the 3-month follow-up among booster users. Further research could explore whether it is possible to optimize ICBT for postsecondary populations by using variations of MI and booster lessons.

## Appendix

Multimedia Appendix 1 Estimated marginal means, 95% CIs, percentage changes, and effect sizes (Cohen *d*) for primary and secondary outcomes by treatment condition using pooled imputations after treatment and at the 1-month and 3-month follow-ups.

## Abbreviations

AUDIT: Alcohol Use Disorder Identification Test
CBT: cognitive behavioral therapy
CQ-3: 3-item Change Questionnaire
DUDIT: Drug Use Disorders Identification Test
GAD: generalized anxiety disorder
GAD-7: 7-item Generalized Anxiety Disorder
ICBT: internet-delivered cognitive behavioral therapy
ITT: intention-to-treat
MI: motivational interviewing
PAF: Perceptions of Academic Functioning
PHQ-9: 9-item Patient Health Questionnaire
REDCap: Research Electronic Data Capture
SDS: Sheehan Disability Scale

## Data Availability

Data are not available because of confidentiality concerns.
