# Supplementary material for: Internet-Delivered Cognitive Behavioral Therapy for Postsecondary Students: Randomized Factorial Trial for Examining Motivational Interviewing and Booster Lessons
Source: J Med Internet Res. 2022 Sep 7;24(9):e40637. doi: 10.2196/40637 (PMC9494224; doi:10.2196/40637)
Supplement: Multimedia Appendix 1 [file jmir_v24i9e40637_app1.docx]

Supplementary Table 1. Estimated marginal means, 95% confidence intervals, percentage changes, and effect sizes (Cohen’s d) for primary and secondary outcomes by treatment condition using pooled imputations at post-treatment, 1-month follow-up, and 3-month follow-up

|  | | **Estimated marginal means** | | | |  | **Percentage changes from pre-treatment** | | |  | **Within-group effect sizes from pre-treatment** | | |
| --- | --- | --- | --- | --- | --- | --- | --- | --- | --- | --- | --- | --- | --- |
|  | | **pre-** | **post-** | **1-month** | **3-month** |  | **to post-** | **to 1-month** | **to 3-month** |  | **to post-** | **to 1-month** | **to 3-month** |
| **Primary outcomes** | | |  |  |  |  |  |  |  |  |  |  |  |
| **PHQ-9** | |  |  |  |  |  |  |  |  |  |  |  |  |
| Standard | | 15.15 (6.68) | 7.88 (4.76) | 8.06 (5.17) | 7.90 (5.82) |  | 48  [40, 56] | 47  [38, 55] | 48  [38, 58] |  | 1.25  [0.89, 1.61] | 1.18  [0.82, 1.54] | 1.15  [0.80, 1.51] |
| MI | | 14.45 (5.96) | 6.16 (3.62) | 6.47 (4.68) | 7.33 (4.87) |  | 57  [51, 64] | 55  [47, 64] | 49  [41, 58] |  | 1.67  [1.28, 2.06] | 1.48  [1.10, 1.86] | 1.30  [0.93, 1.67] |
| Booster | | 14.30 (5.18) | 7.93 (4.28) | 7.36 (4.84) | 7.71 (4.92) |  | 45  [36, 53] | 49  [39, 58] | 46  [37, 55] |  | 1.33  [0.97, 1.70] | 1.38  [1.01, 1.74] | 1.30  [0.93, 1.66] |
| MI/  Booster | | 14.03 (5.17) | 7.66 (4.54) | 7.63 (5.17) | 7.35 (4.62) |  | 45  [37, 54] | 46  [36, 55] | 48  [39, 56] |  | 1.30  [0.93, 1.67] | 1.23  [0.86, 1.60] | 1.35  [0.98, 1.73] |
| **GAD-7** | |  |  |  |  |  |  |  |  |  |  |  |  |
| Standard | | 14.27 (4.81) | 7.19 (4.53) | 7.27 (4.82) | 6.73 (4.77) |  | 50  [41, 58] | 49  [40, 58] | 53  [44, 62] |  | 1.51  [1.13, 1.88] | 1.44  [1.08, 1.81] | 1.57  [1.19, 1.94] |
| MI | | 13.21 (4.76) | 5.19 (2.99) | 5.92 (4.42) | 7.50 (5.77) |  | 61  [55, 67] | 0.55  [46, 64] | 43  [32, 54] |  | 2.01  [1.59, 2.42] | 1.58  [1.19, 1.97] | 1.07  [0.71, 1.44] |
| Booster | | 13.51 (4.84) | 7.16 (4.05) | 6.87 (5.02) | 8.30 (5.60) |  | 47  [39, 55] | 49  [39, 59] | 39  [28, 50] |  | 1.42  [1.05, 1.78] | 1.34  [0.98, 1.70] | 0.99  [0.64, 1.34] |
| MI/  Booster | | 13.82 (4.68) | 7.04 (4.34) | 7.67 (4.87) | 6.80 (5.05) |  | 49  [41, 57] | 45  [35, 54] | 51  [41, 61] |  | 1.49  [1.11, 1.87] | 1.28  [0.91, 1.65] | 1.44  [1.06, 1.81] |
| **PAF** | |  |  |  |  |  |  |  |  |  |  |  |  |
| Standard | | 17.04 (6.88) | 18.99 (7.49) | 20.46 (5.90) | 19.53 (7.08) |  | 15  [0, 30] | 26  [14, 39] | 19  [4, 35] |  | 0.27  [-0.06, 0.60] | 0.53  [0.20, 0.87] | 0.36  [0.02, 0.69] |
| MI | | 17.84 (5.38) | 19.18 (7.84) | 19.57 (6.99) | 19.48 (8.29) |  | 11  [-5, 28] | 14  [-1, 30] | 14  [-4, 31] |  | 0.20  [-0.14, 0.54] | 0.28  [-0.06, 0.62] | 0.23  [-0.11, 0.57] |
| Booster | | 17.46 (6.49) | 19.46 (7.28) | 20.27 (6.36) | 18.85 (7.74) |  | 16  [1, 31] | 22  [8, 37] | 11  [-5, 27] |  | 0.29  [-0.04, 0.62] | 0.43  [0.10, 0.77] | 0.19  [-0.14, 0.52] |
| MI/  Booster | | 17.10 (7.05) | 20.37 (6.57) | 20.78 (6.38) | 20.37 (6.73) |  | 25  [12, 39] | 29  [15, 42] | 25  [12, 39] |  | 0.48  [0.14, 0.82] | 0.54  [0.20, 0.89] | 0.47  [0.13, 0.81] |
| **Secondary outcomes** | | |  |  |  |  |  |  |  |  |  |  |  |
| **SDS** | |  |  |  |  |  |  |  |  |  |  |  |  |
| Standard | | 20.54 (5.80) | 16.24 (7.57) | 13.17 (7.12) | 12.37 (7.69) |  | 21  [12, 30] | 36  [27, 45] | 40  [30, 50] |  | 0.63  [0.30, 0.97] | 1.13  [0.77, 1.48] | 1.19  [0.83, 1.55] |
| MI | | 19.01 (6.67) | 12.49 (6.82) | 11.43 (6.96) | 12.68 (7.76) |  | 34  [25, 44] | 40  [30, 50] | 33  [23, 44] |  | 0.96  [0.60, 1.32] | 1.11  [0.74, 1.47] | 0.87  [0.52, 1.22] |
| Booster | | 19.59 (5.70) | 15.27 (6.29) | 11.83 (6.95) | 12.19 (7.09) |  | 22  [14, 30] | 40  [30, 49] | 38  [29, 47] |  | 0.72  [0.38, 1.06] | 1.21  [0.86, 1.57] | 1.14  [0.79, 1.50] |
| MI/  Booster | | 19.56 (6.77) | 13.89 (6.69) | 12.39 (7.47) | 12.28 (7.17) |  | 29  [19, 39] | 37  [27, 47] | 37  [27, 47] |  | 0.84  [0.49, 1.19] | 1.00  [0.64, 1.36] | 1.04  [0.68, 1.40] |
| **AUDIT^1^** | |  |  |  |  |  |  |  |  |  |  |  |  |
| Standard | | 4.52 (4.28) | 3.64 (4.17) | - | - |  | 20  [-3, 42] | - | - |  | 0.21  [-0.12, 0.54] | - | - |
| MI | | 4.64 (5.04) | 3.07 (3.78) | - | - |  | 34  [14, 54] | - | - |  | 0.35  [0.01, 0.69] | - | - |
| Booster | | 4.06 (3.84) | 3.38 (3.56) | - | - |  | 17  [-7, 40] | - | - |  | 0.18  [-0.15, 0.51] | - | - |
| MI/  Booster | | 4.54 (4.71) | 3.11 (4.09) | - | - |  | 32  9, 54] | - | - |  | 0.32  [-0.02, 0.66] | - | - |
| **DUDIT^1^** | |  |  |  |  |  |  |  |  |  |  |  |  |
| Standard | | 2.93 (6.63) | 2.78 (6.18) | - | - |  | 5  [-44, 55] | - | - |  | 0.02  [-0.31, 0.35] | - | - |
| MI | | 2.48 (5.51) | 2.51 (5.83) | - | - |  | -1  [59, 56] | - | - |  | -0.01  [-0.34, 0.33] | - | - |
| Booster | | 2.87 (5.44) | 1.88 (3.95) | - | - |  | 35  [0, 69] | - | - |  | 0.21  [-0.12, 0.54] | - | - |
| MI/  Booster | | 2.16 (4.71) | 1.37 (3.22) | - | - |  | 37  [-2, 75] | - | - |  | 0.20  [-0.14, 0.53] | - | - |
|  | *Note.* MI = motivational interviewing; PHQ-9 = Patient Health Questionnaire-9; GAD-7 = Generalized Anxiety Disorder-7; PAF = Perceptions of Academic Functioning; SDS = Sheehan Disability Scale; AUDIT = Alcohol Use Disorder Identification Test; DUDIT = Drug Use Identification Test.  ^1^The AUDIT and DUDIT were only administered at pre-treatment and post-treatment, so data is not available for the percentage change and effect sizes at 1-month and 3-month follow-up. | | | | | | | | | | | | |
